# SiameseNet based on multiple instance learning for accurate identification of the histological grade of ICC tumors

**DOI:** 10.3389/fonc.2025.1450379

**Published:** 2025-02-10

**Authors:** Zhizhan Fu, Fazhi Feng, Xingguang He, Tongtong Li, Xiansong Li, Jituome Ziluo, Zixing Huang, Jinlin Ye

**Affiliations:** ^1^ The Quzhou Affiliated Hospital of Wenzhou Medical University, Quzhou People’s Hospital, Quzhou, China; ^2^ Yangtze Delta Region Institute (Quzhou), University of Electronic Science and Technology of China, Quzhou, China; ^3^ Department of Radiology, West China Hospital, Sichuan University, Chengdu, China

**Keywords:** intrahepatic cholangiocarcinoma, histological grade, multiple instance learning, cross-attention mechanism, CT-based diagnostics

## Abstract

**Background:**

After hepatocellular carcinoma (HCC), intrahepatic cholangiocarcinoma (ICC) is the second most common primary liver cancer. Timely and accurate identification of ICC histological grade is critical for guiding clinical diagnosis and treatment planning.

**Method:**

We proposed a dual-branch deep neural network (SiameseNet) based on multiple-instance learning and cross-attention mechanisms to address tumor heterogeneity in ICC histological grade prediction. The study included 424 ICC patients (381 in training, 43 in testing). The model integrated imaging data from two modalities through cross-attention, optimizing feature representation for grade classification.

**Results:**

In the testing cohort, the model achieved an accuracy of 86.0%, AUC of 86.2%, sensitivity of 84.6%, and specificity of 86.7%, demonstrating robust predictive performance.

**Conclusion:**

The proposed framework effectively mitigates performance degradation caused by tumor heterogeneity. Its high accuracy and generalizability suggest potential clinical utility in assisting histopathological assessment and personalized treatment planning for ICC patients.

## 1 Introduction

Primary liver cancer is a malignant tumor that begins in the liver. It was the fourth-commonest cancer in men and the eleventh commonest cancer in women (1). Among the several types of primary liver cancer, hepatocellular carcinoma (HCC) is the most common form of liver cancer in adults, and intrahepatic cholangiocarcinoma (ICC) is the second most common malignancy in the liver. Despite being less common than HCC, the incidence of ICC is on the rise in many countries (2). ICC makes up approximately 10% of all cholangiocarcinomas, and the median survival period for patients is less than 3 years (3).

The histological grade of tumor is closely related to prognosis, tumors with low histological grade often have a better prognosis compared to tumors with high histological grade (4, 5). Therefore, promptly detecting the histological grade of ICC tumors is crucial for patient treatment and prognosis.

Traditionally, the assessment of the histological grade of ICC is based mainly on immunohistochemistry, and the tumor tissue is typically obtained by needle biopsy (6). However, invasive biopsies have several limitations, including sample bias caused by tumor heterogeneity and high costs (7). By contrast, computed tomography (CT) is a real-time and noninvasive method for liver disease diagnosis. With CT, continuous and/or overlapping high-resolution thin-slice (0.75–1.5 mm) images of the entire abdomen can be acquired. Previous studies have suggested that the imaging characteristics of a tumor might be used to help the disease diagnosis (8–11); therefore, using CT images to determine the degree of ICC histological grade appears feasible. However, effectively using CT images to identify the histological grade of ICC remains a challenge.

Deep learning (DL) is a common and efficient method of obtaining knowledge from images because convolutional neural networks (CNNs) have a strong ability to recognize patterns in images (12–15). DL models typically require labels for individual images during training. However, a common challenge arises when working with medical imaging data, specifically CT scans, in which labels are often assigned to entire patients rather than individual slice images. Because CT scans consist of a multitude of slices capturing different anatomical details, the lack of specific labels for each slice complicates the task of imparting granular information to the model. Assigning labels to each CT image slice for training introduces several noisy labels, because different CT slices of the same patient may contain varying lesion areas, and their histological grade may also differ (16). To address this problem, multiple-instance learning (MIL) was introduced, which requires labels only for each patient (17). MIL represents a type of weakly-supervised classification that depends solely on patient-level labels, where each slice is treated as an individual instance, and a single case or patient data comprising multiple slices is treated as a “bag” (18, 19).

Furthermore, CT images typically contain information about the lesion and its surroundings. Environmental information in the background can help identify the histological grade of ICC to a certain extent; however, it also introduces considerable noise. To allow the model focus on to both the lesion and surrounding environment, the original CT images are segmented into images containing the lesion and background environment (hard images) and images containing only the lesion area (easy images) based on a lesion mask provided by the doctor.

DL has been widely used in the field of medical imaging (20–23). The objective of this study was to develop a deep-learning model to predict the histological grade of ICC using CT images. We designed a multiple-instance CNN, called SiameseNet, containing two branches to process the input information of the two modalities. Because determining the correspondence between the two types of images is challenging, we leveraged a cross-attention module to dynamically learn the correlation between hard and easy images (24, 25).

Intrahepatic cholangiocarcinoma (ICC) poses significant diagnostic challenges due to its reliance on invasive biopsies and the heterogeneity of tumor histological grades. This study introduces a novel dual-branch SiameseNet, incorporating Multi-Instance Learning and cross-attention mechanisms, to predict ICC histological grades non-invasively from CT images. By addressing the limitations of current methods, such as sample bias in biopsies and the inability to fully utilize imaging data, this approach has the potential to revolutionize diagnostic workflows and enable more accurate, safer, and faster grading of ICC tumors.

To the best of our knowledge, no research has been reported for predicting the histological grade of ICC by combining MIL and a double-branch CNN. The main contributions of our work are: 1) We propose an MIL method to predict the histological grade of ICC based on CT images. 2) We incorporated two different data modalities in the model training and designed a multiple-instance model with dual branches for this task. 3) To efficiently integrate the information from the two modalities, we implemented a cross-attention mechanism within the model.

## 2 Materials and methods

### 2.1 Patient cohort

**Figure 1:**
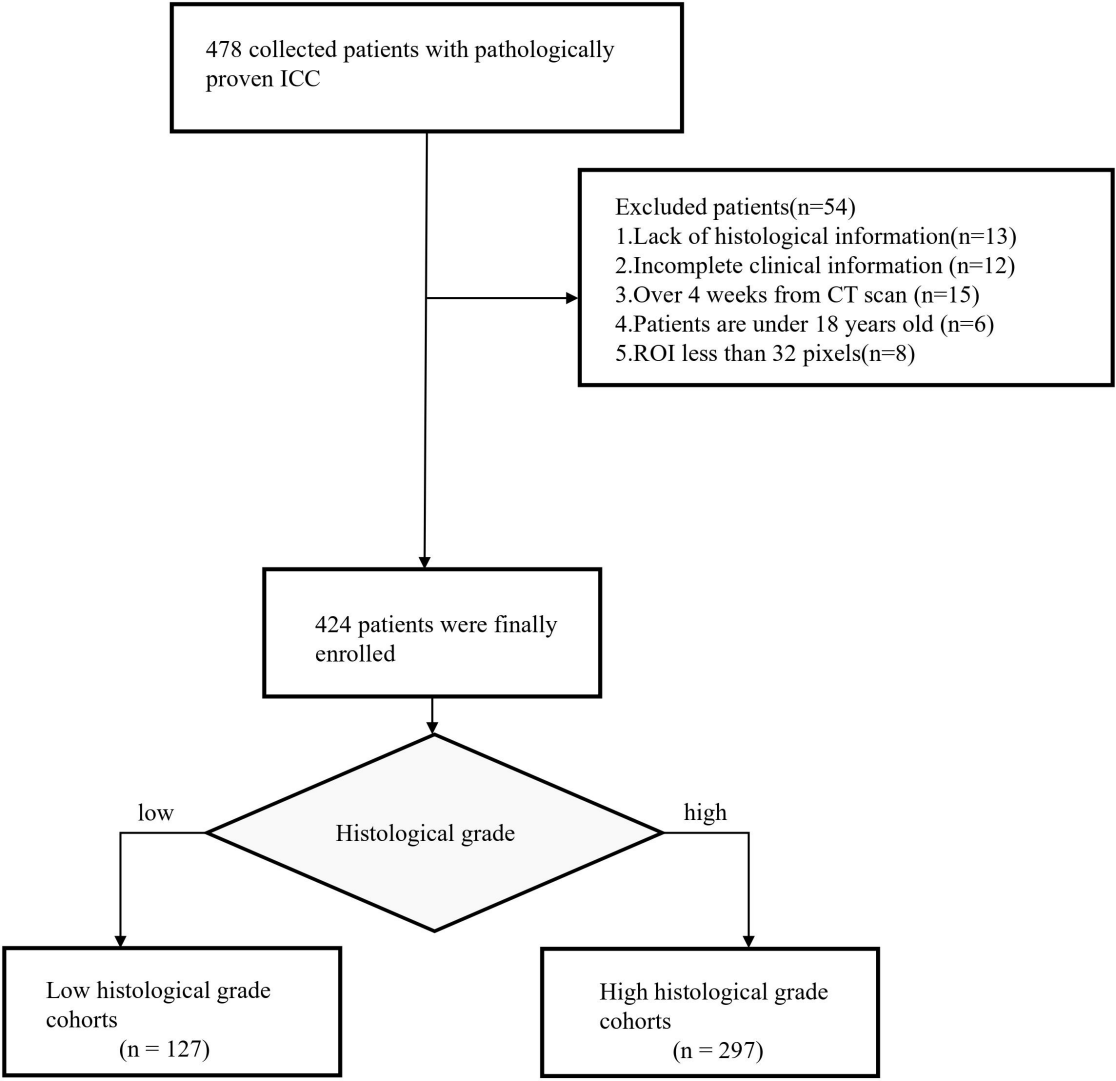
Patient recruitment process.

Intrahepatic cholangiocarcinoma (ICC) is a relatively rare form of primary liver cancer, accounting for only about 10% of all cholangiocarcinomas. This rarity makes it challenging to collect large datasets, even in specialized medical centers. Among the collected cases, only a subset includes detailed histological grading, further restricting the pool of eligible cases for our study. This study was approved by the local ethics review committee(20211343), which waived the requirement for informed consent. The data of a total of 424 patients with ICC were included in the study and met the following criteria: (1) age over 18 years, (2) pathologically confirmed diagnosis of ICC, and (3) complete baseline characteristics, laboratory tests, and tumor pathology records. Details are shown in **Figure 1**.

### 2.2 Histopathological examinations

The surgically resected hepatic specimens were used for the pathological evaluation. Identification of the pathological characteristics were performed by a team of experienced pathologists (each individual with more than 10 years of experience in reading histopathological slices), who were blinded to the CT and clinical results. Tumor differentiation were evaluated and identified as well-differentiated, well-to-moderated differentiated, moderate-differentiated, moderate-to-poor differentiated, poor-differentiated. In this study, well-differentiated, well-to-moderated differentiated, moderate-differentiated were divided into low-histological grade, moderate-to-poor differentiated, poor-differentiated were divided into high-histological grade.

### 2.3 CT images

It is important to preprocess original abdominal CT images to enhance the accuracy of liver lesion detection models. These images typically contain various organs and tissues that can introduce noise and interfere with the model training process. We therefore adopted a two-step preprocessing approach. First, we obtained manual masks from professional doctors to identify the lesion area in each CT slice. These masks served as ground-truth labels for model training. Using this mask information, we extracted two different modalities of image data. The first modality consisted of the lesion area identified by the mask alone. By isolating only the lesion area, we eliminate interference from other irrelevant organs and tissues, allowing the model to focus more accurately on the target region. The second modality involved extending the image outward from the center of the mask to a certain size. In this study, the image size was extended to 128 × 128 pixels. If the lesion area extended beyond this size, the boundaries of the lesion area were preserved. Using this approach, we retained a small area around the lesion outside the intrahepatic lesion. This area helped the model to better distinguish the differences and connections between the lesion and the normal area, thereby improving overall performance. In addition to these preprocessing steps, we performed data augmentation techniques on the images during the training process. This process included techniques such as color jitter (26) to adjust image brightness and contrast, as well as random resizing, cropping, and flipping of the images (RandomResizedCrop and RandomHorizontalFlip) (27) to introduce variety and increase the diversity of the training data. By utilizing these preprocessing techniques and data augmentation methods, we aimed to enhance the performance and accuracy of the liver lesion detection model.

### 2.4 Siamese network

**Figure 2:**
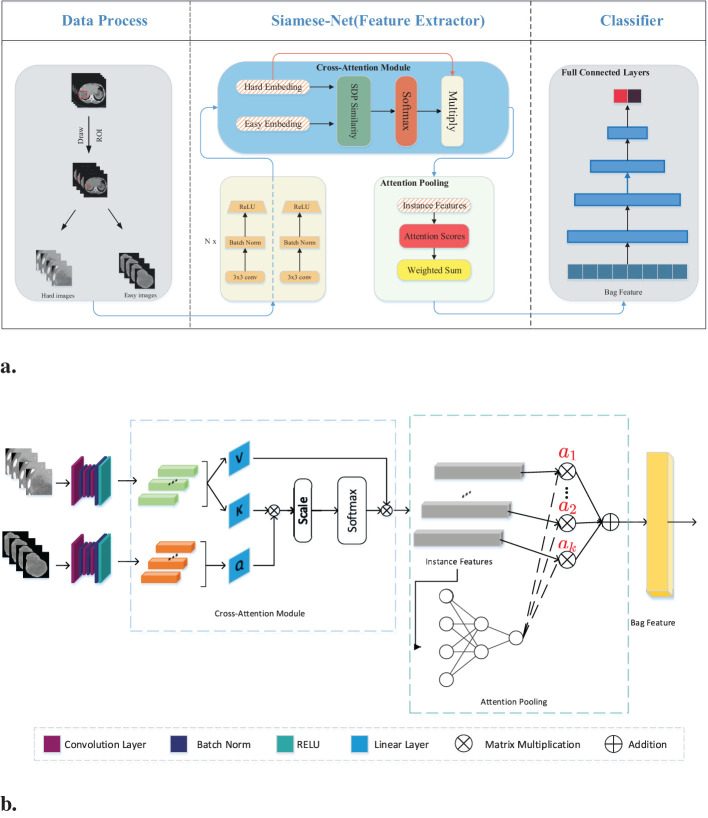
**(A)** Workflow overview including data processing and feature extraction networks: SiameseNet and nonlinear classifier. In the data processing stage, the processed images are the patient’s abdominal CT plain scan slices. From the mask information drawn by the doctor, we extracted the original image into two-modal information. Those containing the lesion area and surrounding environment information are hard images. Easy images include only the lesion area. **(B)** The SiameseNet network structure consists of two independent CNN branches—a cross-attention module and an attention-pooling module. The red color corresponds to attention scores for each instance.

**Figure 3:**
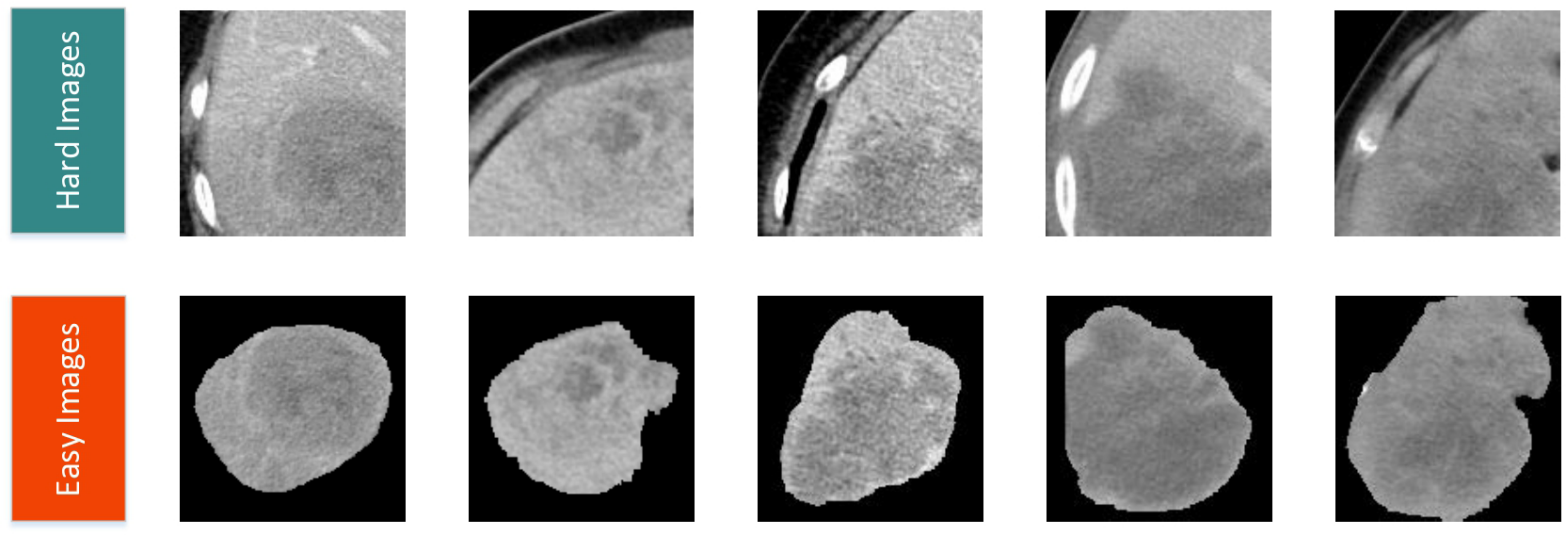
Easy and hard images of patients.

**Figure 2A** illustrates the detailed workflow of the proposed model. The first step is data processing, in which two types of modal information are extracted from the original abdominal CT slices. This extraction is based on the mask information of the lesion area provided by the doctor. The first type of modal information, called hard images, contains environmental information about the lesion area and its surroundings. This type of information is more complex and contains a larger amount of data, making it more challenging for the model to process. The second type, called easy images, includes only the lesion area. As the name suggests, this type of information makes it easier for the model to identify and process. **Figure 3** are some easy and hard images of patients. To enhance the model’s generalization performance, we utilized data augmentation techniques to enrich the patterns of the training samples. This approach ensures that the model can learn effectively from various input variations.

Our network model is a two-branch multi-instance network model, as shown in **Figure 2B**. The input to the model includes all the CT image slices of each patient. These images are first fed into a CNN for feature extraction. Because the two modalities (hard and easy images) contain different amounts of information and complexities, the CNN parameters of the two branches are updated independently. They do not affect each other during the training process. To further improve the model’s ability to identify the lesion area, we introduced a cross-attention mechanism. This mechanism calculates the cross-attention between the images of the two modalities. This approach enables the model to focus on the confidence of the lesion area while learning the relationship between the lesion area and the surrounding environment. This cross-attention mechanism enhances the model’s performance. After passing through the cross-attention module, the feature vectors of the two modalities are fused into several instance features. These instance features are then merged into a package feature using the attention aggregation module in MIL, and at the end of our network, the package features are passed through a nonlinear classification head to obtain the final classification result, which represents the model’s ability to predict the presence or absence of the targeted condition on abdominal CT scans. Besides, we implemented early stopping during training to prevent overfitting to the training set.

### 2.5 Cross-attention module

We introduced a cross-attention mechanism to improve recognition of the lesion area in our network. This mechanism utilizes information from both the lesion area and the surrounding environment to enhance the model’s understanding. The model takes two modal inputs, each containing information about the lesion area but with different levels of complexity and amounts of information. The model was made to focus more on the lesion area by leveraging environmental information using a CNN to extract feature vectors from both easy and hard images. The feature vector extracted from the easy images was treated as the query vector, and the feature vector from the hard images was used as the key-value vector.

To calculate the similarity score, we measured the similarity between the query vector and each key vector. This similarity score helps determine the importance of each key-value vector. A higher similarity score implies that the key-value vector contains more relevant information about the lesion area. From the similarity scores, we subsequently calculated a weighted sum of the corresponding value vectors. The weighted sum represents an enhanced representation of the lesion area. By incorporating this cross-attention mechanism, we ensure that the model focuses more on the lesion area by taking advantage of environmental information. This approach can improve recognition accuracy and provide a better understanding of the lesion area using the model, mathematically expressed as:


(1) $$\begin{array}{c} Cross-Attention\left(Q,K,V\right)\\ =softmax\left(SDP-Similarity\left(Q,K\right)\right)V \end{array}$$

where SDP−Similarity(Q,K) is the scaled dot product similarity calculation. By dividing the dot product result by the square root of the dimensionality, we scale down the similarity score, which can be written as:


(2) $$SDP-Similarity\left(Q,K\right)=\frac{QK^{T}}{\sqrt{d_{k}}}$$

where $d_{k}$ is the dimension of the feature vector in the matrices *Q* and *K*. This scaling prevents the dot product result from becoming too large, which in turn helps avoid overly small gradients during the softmax operation. We ensure that the similarity scores are well-scaled and facilitate smooth and effective backpropagation in the training process of the model using the scaled dot product similarity calculation. This approach allows for better adjustment of the model parameters and ultimately improves its performance in recognizing and understanding the lesion area.

### 2.6 Multi-instance learning

MIL is a machine learning paradigm designed to handle scenarios where labels are assigned to groups of instances (called “bags”) rather than to individual instances. This framework is particularly useful when instance-level labels are unavailable, noisy, or difficult to obtain. For our task, each patient has several CT slices. We only have the histological grade label of each patient. It is very difficult to obtain the histological grade label of each CT slice, so we decided to use MIL to train our data.

The training data for this study comprised CT slice images of patients, in which each patient had multiple CT slices. These slices mostly contained lesion areas; however, the histological grade of these lesion areas may vary owing to tumor heterogeneity. To avoid introducing noise during model training, we utilized the MIL technique. MIL is a weakly supervised method that differs from traditional learning methods in the composition of training instances. In traditional supervised learning, each instance is represented by $\left(X_{i},\;Y_{i}\right)$ , where $X_{i}$ is the instance and $Y_{i}$ is the corresponding label. However, in MIL, training samples are no longer a single instance, but “bags” of instances. Each package is in the form of $\left(\{X_{i1},\;X_{i2},\ldots ,\;X_{in}\},\;Y_{i}\right)$ , where the number of instances in each package can vary and the label of each instance is unknown. The goal of MIL is to train a classifier using these packages to predict the labels of the unknown packages. Generally, a pooling module exists to fuse the instance features into the bag feature. Max pooling and average pooling are two commonly used pooling methods. Max pooling can be expressed as:


(3) $$z=\underset{k=1,\ldots ,K}{\max}h_{k}$$

and average pooling as:


(4) $$z=\frac{1}{K}\sum_{k=1}^{K}h_{k}\;$$

Where $h_{k}$ is the kth instance feature.

In our case, we considered the set of slices from all the CT images of each patient as a package and used the histological grade of ICC as the label for the package. To fuse the instance features extracted by the cross-attention module into the final bag features, we use a pooling method based on the attention mechanism. Compared to max pooling and average pooling, this pooling method improves the effectiveness and interpretability of multi-instance learning. Another benefit is that it is differentiable and can be trained through neural networks. The attention weights from the attention pooling module allow us to understand which instances contribute more to the label. Let $H=\;\left\{h_{1},\ldots ,h_{k}\right\}$ represent a package with K instances, where $h_{i}$ is the embedding obtained from the ith instance through the feature extraction network. The attention-based MIL pooling is expressed as follows:


(5) $$z=\sum_{k=1}^{K}a_{k}h_{k},\;a_{k}=\frac{\text{exp}\{w^{T}\text{tanh}\;(Vh_{k}^{T})\}}{\sum_{j=1}^{K}\text{exp}\{w^{T}\text{tanh}\;(Vh_{j}^{T})\}\;}$$

After fusing the bag features, they are input into a nonlinear fully connected network to obtain the final prediction result. The cross-entropy loss function is used, which measures the difference between the predicted probabilities and actual labels. It can be expressed as follows:


(6) $${ℒ}_{CE}=-\sum \left(y\text{log }\hat{y}+\left(1-y\right)\text{ log }(1-\hat{y})\right)$$

where *y* represents the real label and $\hat{y}$ represents the predicted probability value. This loss function quantifies the dissimilarity between the predicted and actual labels.

### 2.7 Hyperparameter optimization

We utilized a grid search approach to systematically evaluate a range of hyperparameters, including learning rate, batch size, number of epochs. For certain hyperparameters (e.g., optimizer type), manual tuning was conducted based on prior studies and preliminary experiments. Hyperparameters were optimized using the validation accuracy as the primary metric, while ensuring a balance between training and validation performance to minimize overfitting. Here are our final hyperparameter values:Learning rate:5e-4 (with decay factor of 0.1 every 10 epochs). Batch size: 128 (For MIL, batch size is 1). Optimizer: Adam with $\beta_{1}$ =0.9, $\beta_{2}$ =0.999. Number of epochs: 200 (early stopping was applied if validation accuracy plateaued for 10 consecutive epochs).

### 2.8 Computational requirements and cost-benefit analysis

The model was trained and tested on a workstation equipped with an NVIDIA RTX 3090 GPU (24 GB RAM). Training the model on the full dataset (381 training samples) required approximately 6 hours, including data augmentation and optimization steps. For a single patient (bag of CT slices), the inference process, including feature extraction, cross-attention computation, and classification, takes approximately 10 seconds on the same GPU hardware.

The proposed SiameseNet model offers significant advantages over traditional biopsy-based methods for ICC histological grade assessment. It is non-invasive, time-efficient, and cost-effective, providing results in seconds without the risks associated with invasive procedures. While initial deployment costs, such as computational infrastructure, are required, these are offset by the reduction in biopsy and pathology expenses.

## 3 Results

### 3.1 Patient characteristics

**Table 1** shows the demographic and clinical characteristics of the patients used to train and test the SiameseNet model. According to the size of the ICC patient dataset, we randomly divided it into mutually exclusive training and testing sets, using a classic holdout strategy (28) with an allocation ratio of 9:1. We used a random stratified sampling method to split the dataset. This approach ensures that the distribution of key attributes, particularly the histological grade (low-grade vs. high-grade), is consistent between the training and testing sets. Specifically, we stratify the dataset based on histological grade, then randomly allocate 90% of cases from each grade category to the training set and the remaining 10% was assigned to the testing set.

**Table 1:** The statistics of ICC patients in the training and testing cohorts.

Attribute	Training Cohort			Testing Cohort		
	High-HG	Low-HG	*p* Value	High-HG	Low-HG	*p* Value
**Age**			0.027			0.781
Mean (SD)	56.39 (10.64)	59.06 (9.81)		59.57 (10.43)	58.85 (11.24)	
**Gender**			0.975			0.084
Male (%)	142 (0.53%)	60 (0.53%)		18 (0.6%)	4 (0.31%)	
Female (%)	126 (0.47%)	54 (0.47%)		12 (0.40%)	9 (0.69%)	
**CEA**			0.509			0.218
>5 (%)	75 (0.28%)	32 (0.28%)		6 (0.2%)	1 (0.08%)	
**INR**			0.511			0.516
Mean (SD)	1.04 (0.27)	1.01 (0.10)		1.00 (0.09)	0.98 (0.09)	
**CA199**			0.286			0.720
>37 (%)	157 (0.59%)	78 (0.68%)		23 (0.77%)	9 (0.54%)	
**FIB**			0.256			0.290
>4 (%)	67 (0.25%)	22 (0.19%)		9 (0.3%)	3 (0.23%)	
**AFP**			0.160			0.435
Mean (SD)	22.06 (104.28)	15.37 (79.22)		3.17 (2.04)	3.26 (2.49)	
**ALT**			0.760			0.072
>55 (%)	43 (0.16%)	19 (0.17%)		7 (0.23%)	1 (0.08%)	
**ALP**			0.717			0.059
>129 (%)	79 (0.3%)	42 (0.37%)		13 (0.43%)	2 (0.15%)	
**TBIL**			0.472			0.947
>22.24 (%)	25 (0.09%)	18 (0.16%)		3 (0.1%)	2 (0.15%)	
**GGT**			0.611			0.076
Mean (SD)	116.32 (160.88)	144.88 (213.84)		162.70 (193.61)	142.23 (311.59)	
**HBsAg**			0.708			0.437
Positive (%)	74 (0.28%)	34 (0.3%)		8 (0.27%)	2 (0.15%)	
Negative (%)	191 (0.72%)	80 (0.7%)		22 (0.73%)	11 (0.85%)	
**HBeAg**			0.605			0.365
Positive (%)	7 (0.03%)	2 (0.02%)		2 (0.07%)	0 (0)	
Negative (%)	258 (0.97%)	112 (0.98%)		28 (0.93%)	13 (100%)	

The interpretation of data includes CEA, carcinoembryonic antigen (ng/mL); INR, international normalized ratio; CA199, carbohydrate antigen 19-9 (U/ml); FIB, fibrinogen (g/L); AFP, alpha-fetoprotein (ng/mL); ALT, alanine aminotransferase (IU/L); ALP, alkaline phosphatase (U); TBIL, total bilirubin (µmol/L); GGT, γ-glutamyl transpeptidase (g/L); HBsAg, hepatitis B surface antigen; HBeAg, hepatitis B e antigen; SD, standard deviation; HG, Histological grade.

Bold text indicates metric names (e.g., age, gender, CEA, INF) for patients statistics.

The method we used to calculate the P-value is the Mann-Whitney U test, also known as the Mann-Whitney-Wilcoxon test or the rank-sum test, is a non-parametric statistical method used to compare two independent samples. Unlike the t-test, the Mann-Whitney U test does not require the data to follow a normal distribution, making it suitable for situations where the sample distribution is unknown or cannot be assumed to be normal. The test compares the ranks of the two samples to determine if they come from the same distribution. In this study, the data is separated into two groups based on histological grade, and the ranks of the combined samples from both groups are calculated, followed by the sum of the ranks for each group. Then the statistics U for each of the two groups are computed, and the smaller value is selected as the test statistic U.


(7) $$U=R-\frac{N\;\cdot \;\left(N+1\right)}{2}$$

where N is the number of samples and R is the rank sum of the samples.

Given that the sample size exceeds the upper limit of 20 for the exact distribution table statistics of the Mann-Whitney U test, the normal distribution is employed to approximate the conversion of the statistic U into the standard statistic Z.


(8) $$Z=\frac{U-\mu_{U}}{\sigma_{U}}$$

where $\mu_{U}$ is the expected value of U and $\sigma_{U}$ is the standard deviation of U:


(9) $$\begin{array}{c} \mu_{U}=\frac{N_{High-HG}\cdot N_{Low-HG}}{2},\\ \sigma_{U}=\sqrt{\frac{N_{High-HG}\cdot N_{Low-HG}\cdot \left(N_{High-HG}+N_{Low-HG}+1\right)}{12}} \end{array}$$

The corresponding p-value is obtained using the calculation formula for a two-sided test.


(10) p=2·(1−Φ(|Z|))

where Φ(|Z|) is the cumulative probability of the standard normal distribution.

The proportions of patients with a low histological grade in the training and testing cohorts were 29.9% and 30.2%, respectively. There was no significant difference for gender (train: p = 0.975; test: p = 0.084), CEA (train: p = 0.509; test: p = 0.218), or INR (train: p = 0.511; test: p = 0.516) between the two cohorts, but the prevalence of age appearance was significantly higher (p = 0.027) in the training cohort. However, in the training cohort, there was indeed a significant age difference between patients with low histological grade and those with high histological grade. Specifically, 69.3% (185) of high histological grade cases and 80.7% (92) of low histological grade cases involved patients over the age of 50 years.

This disparity can be explained by the fact that the incidence of ICC is generally higher among individuals aged over 50 years. As a result, a larger proportion of the cases collected in our study naturally fell into this age group. Additionally, it is important to note that low histological grade cases are relatively rare. Consequently, a higher prevalence of low histological grade ICC cases was observed among individuals over 50 years of age as well. Apart from this factor, baseline characteristics and laboratory features were not significant between Low-HD and High-HD patients (ALL p >0.05).

### 3.2 Performance

**Table 2** presents a performance comparison between our SiameseNet model and several other well-established models, including ResNet (29), VisionTransformer (30), SwinTransformer (31), and ConvNext (32). These models encompass a combination of traditional CNNs as well as more recent transformer architecture networks. We conducted a thorough analysis of their variations in accuracy (ACC), area under the curve (AUC), sensitivity (SE), and specificity (SP). The findings indicate that our model demonstrates a notable enhancement of 6.9% and 24.9% in the ACC and AUC, respectively, compared with the baseline model ResNet34. This improvement confirms the efficacy of our model in enhancing predictive accuracy while maintaining superior overall performance without excessive bias towards any particular prediction type.

**Table 2:** Performance comparison of our model with several classic models, including the CNNs ResNet and ConvNext, and the transformer networks VisionTransformer and SwinTransformer.

Model	Training Cohort				Testing Cohort			
	ACC	AUC	SEN	SPEC	ACC	AUC	SEN	SPEC
ResNet34	67.7%	75.6%	79.8%	62.5%	79.1%	61.3%	53.8%	90.0%
VIT	58.5%	55.1%	48.2%	62.9%	76.7%	74.1%	84.6%	73.3%
SwinTransformer-Tiny	69.6%	54.1%	22.8%	89.5%	76.7%	54.1%	46.2%	90.0%
ConvNext	63.5%	48.8%	26.3%	79.4%	79.1%	70.0%	53.8%	90.0%
SiameseNet (Ours)	96.3%	99.2%	95.6%	97.2%	**86.0%**	**86.2%**	**84.6%**	86.7%

ACC, accuracy; AUC, area under receiver operating characteristics curve; SEN, sensitivity; SPEC, specificity.

Bold values highlight the highest scores achieved by the proposed model across comparative experiments (e.g., performance metrics such as AUC or accuracy).

During the training process, we monitored the performance of the model on the validation set. Training was halted as soon as the validation accuracy began to decline, indicating potential overfitting. This approach prioritizes the model’s generalization ability over achieving the highest possible accuracy on the training set. If training had continued beyond the early stopping point, the model’s accuracy on the training set could have reached significantly higher levels. However, this would likely have come at the cost of overfitting, which would reduce the model’s performance on unseen data. By stopping training at an optimal point, the model avoids overfitting while maintaining robust performance on the testing set. The slightly lower accuracy on the training set compared to the testing set reflects this deliberate strategy.

We also compared our cross-attention-based MIL method with other classical MIL methods. The results show that our method significantly outperforms other MIL methods. The compared MIL methods include Mean-MIL, based on average pooling; Max-MIL, based on max pooling (33, 34); attention-based MIL (AB-MIL), based on the attention mechanism (35); and TransMIL, based on the transformer architecture (36). **Table 3** shows the performance of these methods. Compared with the AB-MIL method, our method achieved a 16.2% improvement in ACC and a 21.1% improvement in AUC, indicating that combining data from two different modalities using a cross-attention mechanism can effectively enhance the predictive performance of the model.

**Table 3:** Performance comparison of different MIL methods.

Model	Training Cohort				Testing Cohort			
	ACC	AUC	SEN	SPEC	ACC	AUC	SEN	SPEC
Mean-MIL	96.1%	99.1%	94.6%	97.4%	81.4%	76.8%	61.5%	90.0%
Max-MIL	95.8%	98.7%	94.4%	97.6%	76.7%	57.7%	23.1%	100.0%
AB-MIL	88.5%	94.8%	91.5%	85.0%	69.8%	65.1%	38.5%	83.3%
TransMIL	68.0%	72.6%	75.3%	61.0%	76.7%	53.3%	30.8%	96.7%
CAB-MIL (Ours)	96.3%	99.2%	95.6%	97.2%	**86.0%**	**86.2%**	**84.6%**	86.7%

ACC, accuracy; AUC, area under receiver operating characteristics curve; SEN, sensitivity; SPEC, specificity.

Mean-MIL and Max-MIL are two straightforward MIL methods. AB-MIL and TransMIL are currently widely used classical multiple-instance learning models. Cross-attention-based MIL is the method proposed in this paper.

Bold values highlight the highest scores achieved by the proposed model across comparative experiments (e.g., performance metrics such as AUC or accuracy).

**Figure 4:**
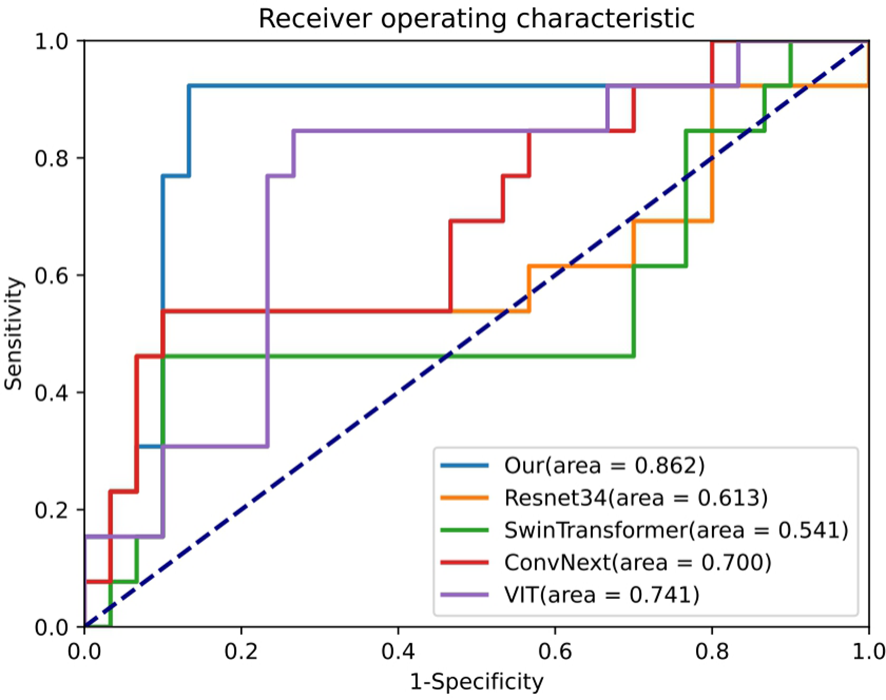
Comparison of ROC curves generated by different networks within the testing cohort. ResNet34, SwinTransformer and ConvNext are popular deep learning models, and Our (AUC=0.862) is the performance of the proposed SiameseNet network.

**Figure 5:**
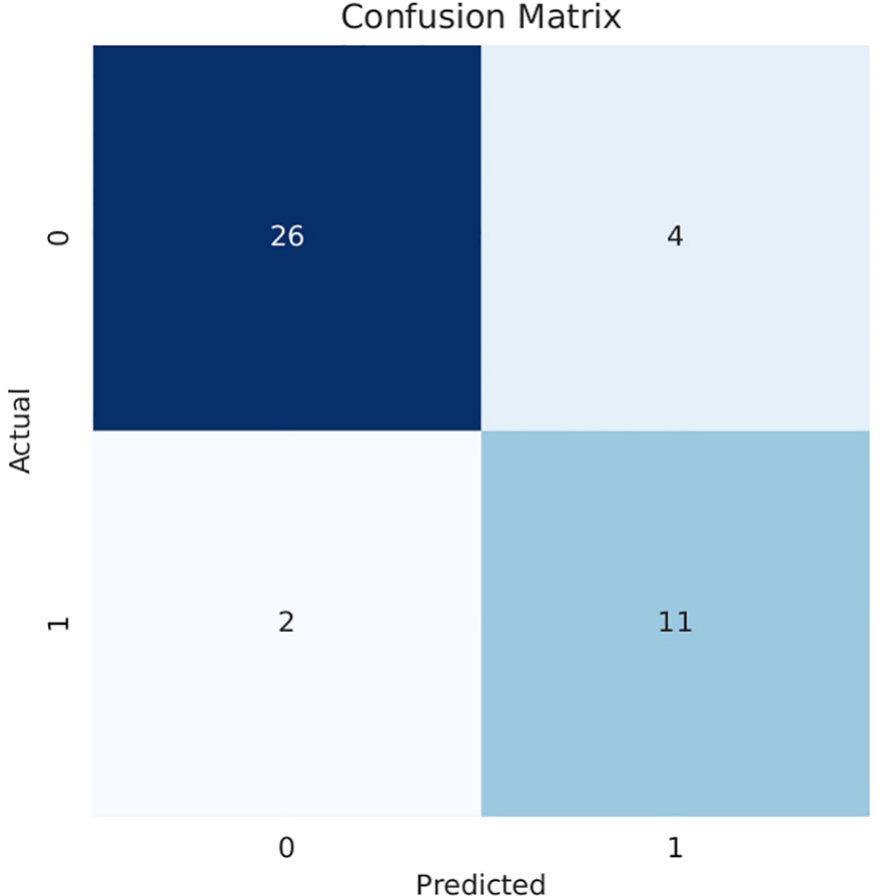
Confusion matrix obtained in the testing cohort.0: High histological grade;1: Low histological grade.

Our model also demonstrated notable enhancements in the receiver operating characteristic (ROC) curve performance in the test cohort compared to other prevalent deep-learning models, as shown in **Figure 4**. And **Figure 5** is the confusion matrix obtained by our model in the test cohort.

### 3.3 Deep learning feature analysis

**Figure 6:**
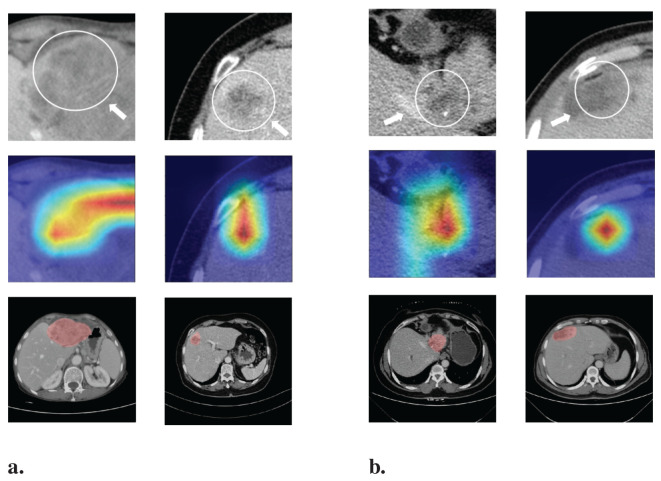
Visualization of the CAM generated by the last convolution layer. Red color denotes high attention values, and blue color denotes low attention values. Subfigure **(A)** shows the CAM of ICC High-HG patient, and subfigure **(B)** shows the CAM of ICC Low-HG patient. HG, Histological grade.

**Figure 7:**
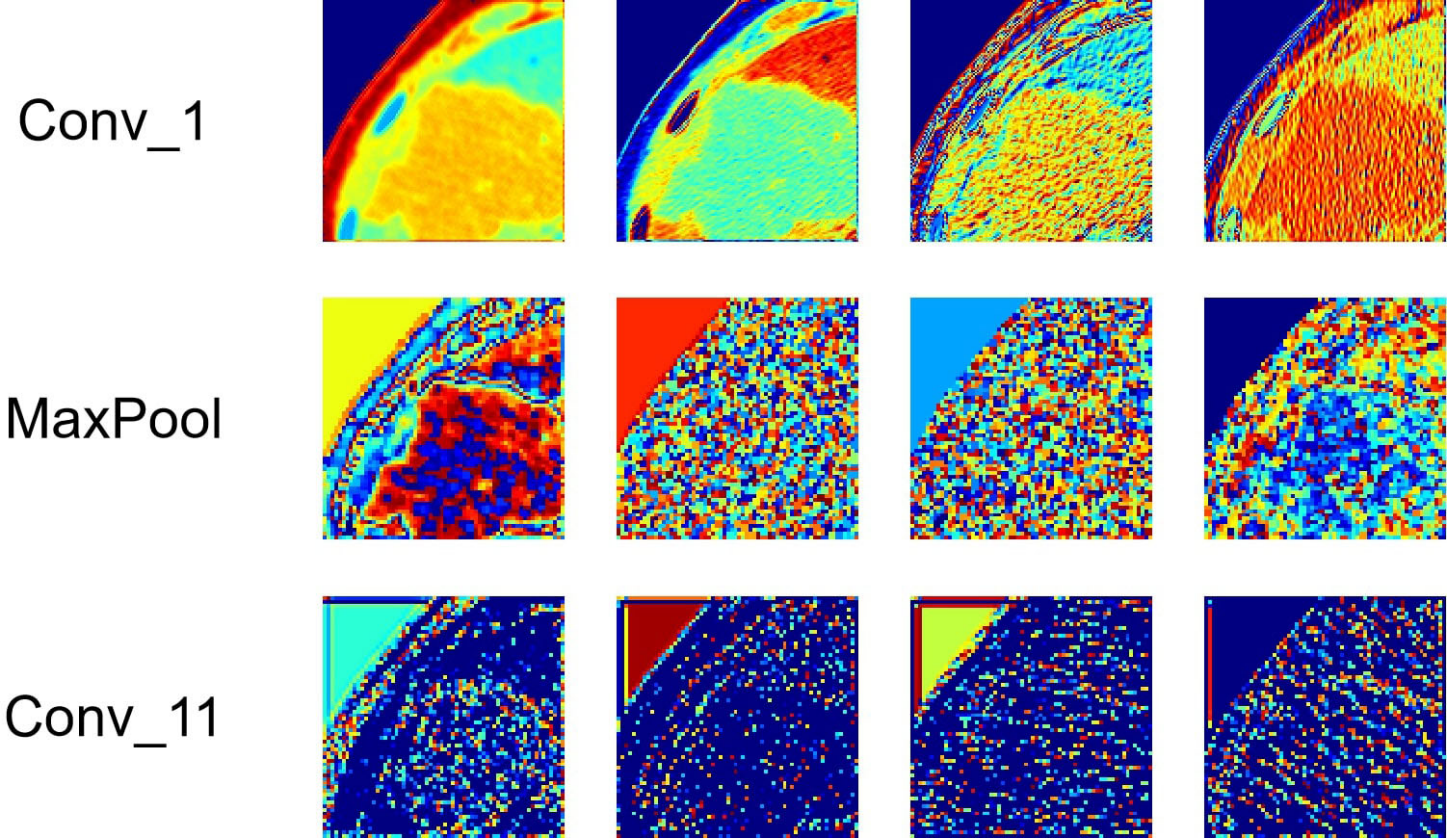
Visualization of the feature maps learned from the convolution of the feature extractor. Each layer of the model includes hundreds of filters, and only four of them are depicted in the figure.

Deep-learning models can automatically extract features for inference by learning the mapping between CT images and the histological grade of ICC. However, because deep-learning models are “black boxes,” we do not know how the model’s inference process is performed. Therefore, it is necessary to use certain methods to increase the interpretability of deep-learning models. In this study, we selected Class Feature Map and Activation Map (CAM) (37, 38) to visualize the feature maps (39) of the model during the inference process and the regions related to lesions to verify the reliability of the model. **Figure 6** shows the attention regions of the model. For CT images, this class activation map represents the importance of various regions learned by the deep-learning model, where the red activation area is more important than the other areas because it is the region signifying more focus by the deep-learning model. From the original image, we can clearly see that the area that the model focuses on is exactly where the tumor is located, which shows that the model uses the information contained in the CT image of the tumor for identification and it can be found that the area that the model focuses on is highly overlapped with the area demarcated by the doctor. **Figure 7** shows the feature maps generated by the feature-extraction part of the model. The shallower convolutional layers learn simple and obvious features (e.g., Conv_1), and the MaxPool layer further amplifies these features, whereas the deeper convolutional layers learn more abstract features (e.g., Conv_11). With the increase in the network depth, the features learned by the model become more abstract and have an increased relationship to the histological grade of ICC. This process is very similar to the process of doctors recognizing images, and it also helps us understand how the model learns.

**Figure 8:**
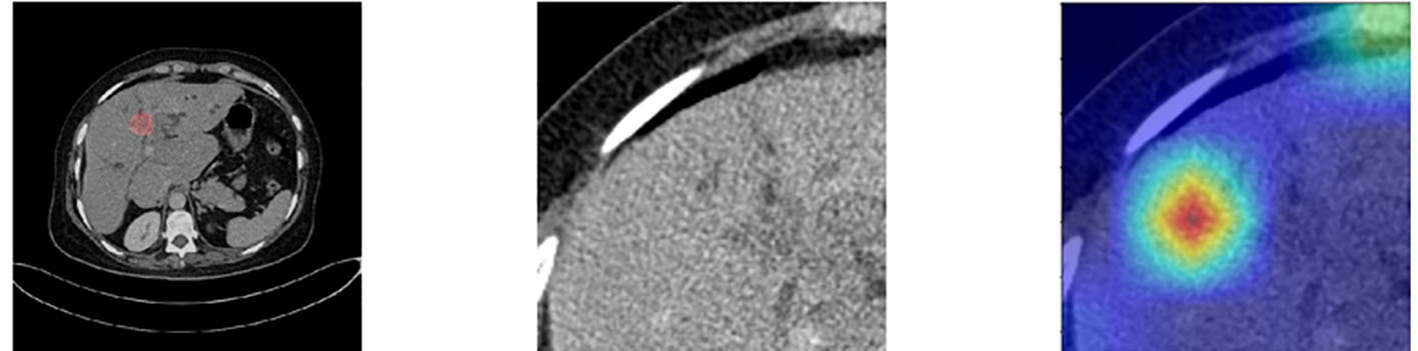
Comparison of the lesions annotated by doctors and the model’s focus areas for patients with prediction failure.

At the same time, we also observed some failure cases. **Figure 8** is a comparison of the lesions annotated by the doctor and the model’s attention area for patients whose model predictions were wrong. We can see that the model mistakenly focused on these areas instead of the actual lesion area. This is an important reason for the model’s recognition errors. How to enhance the model’s recognition ability for this type of atypical images is a focus of our future work.

### 3.4 Ablation study

In this study, we used two modalities of image information for fusion training: images containing only the lesion area (easy images) and images containing both the lesion area and environmental information (hard images). To validate the effectiveness of this method, we compared the training performance using only a single modality of images, either easy or hard images. When using the single-modality image data, we trained the baseline AB-MIL model. **Table 4** presents the model performance using image inputs from different modalities. The results indicate that our multimodal input can effectively improve the recognition accuracy of the model; compared to the model using only easy images, our AUC score also increased by 29.4%.

**Table 4:** Comparison of performance with different modal inputs, with Multi-Modal training method used herein combines easy and hard images.

Model Input	Training Cohort				Testing Cohort			
	ACC	AUC	SEN	SPEC	ACC	AUC	SEN	SPEC
Hard Image	88.5%	94.8%	91.5%	85.0%	69.8%	65.1%	38.5%	83.3%
Easy Image	60.4%	60.4%	57.6%	63.2%	72.1%	52.8%	23.1%	93.3%
Multi-Modal	96.3%	99.2%	95.6%	97.2%	**86.0%**	**86.2%**	**84.6%**	86.7%

ACC, accuracy; AUC, area under receiver operating characteristics curve; SEN, sensitivity; SPEC, specificity.

Bold values highlight the highest scores achieved by the proposed model across comparative experiments (e.g., performance metrics such as AUC or accuracy).

**Figure 9:**
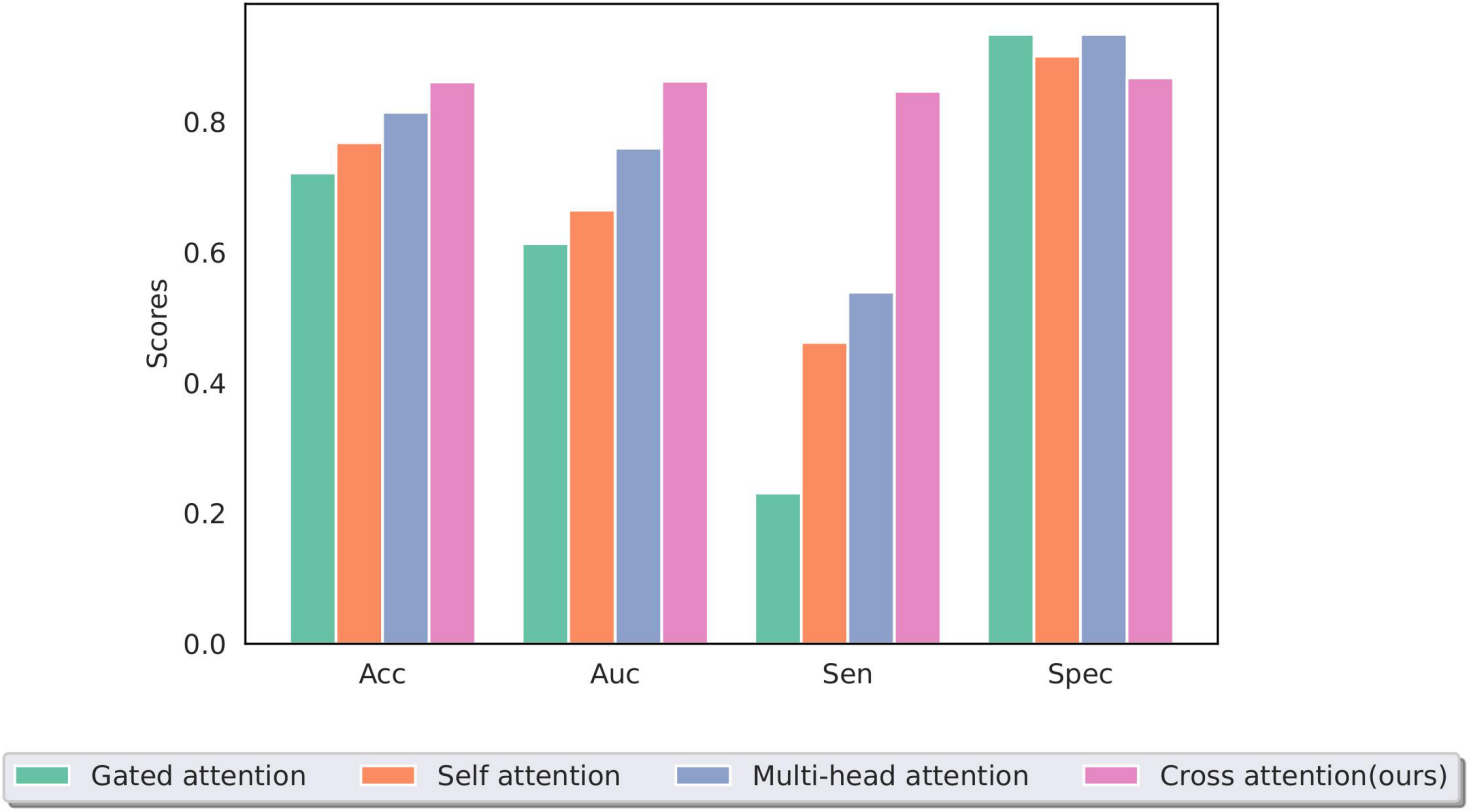
Performance of different multiple-instance fusion methods; cross-attention is used in this study.

The effective and noise information contained in the two modal images differed. To focus the model on the lesion area and reduce the interference of noise information, a cross-attention mechanism was used to fuse the information extracted from the two modal images for training. **Figure 9** shows a performance comparison of our method with commonly used pooling methods in other MIL models, including gated attention (40), self-attention, and multi-head self-attention (41). The experimental results demonstrate that our method can better integrate information from two modal images, effectively improving the model’s performance.

## 4 Discussion

In this study, we constructed SiameseNet, a model based on dual-branch multimodal inputs and MIL. We also utilized a cross-attention mechanism to integrate input information from multiple modalities, enabling the network to predict the histological grade in ICC patients using CT images. Additionally, we found that there were no significant differences in clinical indicators between the ICC high histological grade patient group and the ICC low histological grade patient group, both in the training and testing cohorts. The experimental results indicate that our model is an advanced model that can effectively predict the histological grade of tumor in ICC patients. This method has the potential to assist doctors in assessing the histological grade of tumor in patients with ICC in clinical practice.

The SiameseNet model developed in this study achieved good performance in predicting the histological grade of ICC, with all indicators showing improvements compared to the other methods (ACC = 86.0%, AUC = 86.2%, SEN = 84.6%, SPEC = 86.7%). The performance improvement of the model results mainly from the following aspects: (1) Adopting an MIL training method using all CT slices of a patient as package input to the network for training, effectively alleviating the performance degradation caused by the heterogeneity of tumors. (2) Adopting a dual-branch network structure, with the training data input consisting of two types of CT images containing different amounts of information. This approach ensures that the model does not ignore the effective information in the surrounding environment of the tumor area, which may help with the model’s predictions while allowing the model to focus more on the tumor area without being disturbed by noise in the environmental information. (3) Using a cross-attention mechanism to integrate multimodal information. The traditional pooling method in MIL is not effective for multimodal inputs in this study, whereas the cross-attention mechanism can calculate the similarity between different modal image slices, enabling the model to focus on instances that are most helpful for predictions, thus improving the model’s performance.

MIL is a common deep-learning method for processing medical images. Typically, the original medical images are divided into individual slice images for model training. However, because these slices are obtained from the original 3D images, the sizes of the tumor areas they contain and the histological grade may vary. Multi-instance learning treats all slices as inputs to the model, allowing the model to automatically learn which slices are more important and the relationships between slices. This approach effectively alleviates the decrease in training accuracy caused by tumor heterogeneity. In this study, we adopted an MIL method. We input the information of two modal images into the two branches of the model. Compared with other popular multi-instance methods, such as AB-MIL and TransMIL, our model showed a significant performance improvement (ACC increased by 16.2%, AUC increased by 21.1%). This result indicates that our model has a higher classification accuracy and better predictive results.

The pooling function is a crucial part of multi-instance learning; an effective pooling function enables the model to learn better features and improve its performance. Conventional MIL pooling functions include mean pooling, max pooling, and a series of pooling functions based on an attention mechanism. Mean pooling and max pooling, two of the most intuitive ideas, apply the mean or max operation to the extracted instance features to obtain the final bag feature. This method is mostly effective, but not as effective for medical images, where certain critical slices play a greater role in predicting the image. For this reason, we adopted an attention-mechanism-based pooling approach in this study. Attention-based pooling allows the model to automatically recognize more important instances in the bag. However, previous methods were designed only for a single input and did not effectively learn the relationship between the features extracted by the two branches of the model. Therefore, we adopted a cross-attention mechanism that calculates the similarity between instances from different branches using the features extracted from one branch as the query vector (in this study, images containing only the lesion area) and the features of the other branch as key-value pair vectors. Consequently, the model can fully learn the associative relationships between instances from both branches, ultimately improving its predictive accuracy. The experimental results also show that our pooling method significantly improves the model’s performance, with an increase of 13.9% in ACC and 24.9% in AUC compared with the other methods.

The model could be incorporated into existing clinical workflows, such as assisting radiologists in assessing ICC histological grades based on CT scans or serving as a decision-support tool to provide supplementary insights during diagnosis. The model also have the potential for deploying in real-time clinical scenarios, such as automated processing of CT scans to provide immediate grade predictions alongside radiologists’ assessments. But there are still many challenges, it is important to standardize imaging protocols across institutions to ensure consistent model performance and we should improve the model’s interpretability to build clinician trust in its predictions.

The proposed model has the potential to significantly impact patient management by providing a non-invasive, accurate, and rapid method for predicting ICC histological grades. This can improve diagnostic precision, guide personalized treatment planning, and reduce the need for invasive biopsies. For instance, patients with high-grade tumors identified by the model can be prioritized for systemic therapies, while those with low-grade tumors can benefit from less aggressive approaches. Additionally, the model’s rapid inference enables timely decision-making, critical for managing aggressive cancers. Future studies will focus on validating these impacts in prospective clinical settings.

Our study has some limitations. First, our data were obtained solely from a single medical center, which resulted in a smaller and more localized patient sample. This may introduce biases due to the homogeneity of the patient population, medical practices, or imaging protocols. As such, the findings may not fully reflect the diversity of ICC cases encountered in different clinical settings. Therefore, in future research, we plan to collect data from multiple centers to enhance the generalization performance of the model. Second, the learning and inference processes of the model were not visible and lacked strong interpretability. Despite our efforts to provide explanations through visualization, a gap still exists that must be addressed before the model can be integrated into actual clinical practice. While the proposed model demonstrates promising performance, several limitations must be considered for clinical implementation. These include the single-center nature of the study, variability in imaging protocols, and the need for computational infrastructure in healthcare facilities. Additionally, interpretability challenges and regulatory requirements could impact adoption. Future efforts will focus on multi-center validation, enhancing model explainability, and optimizing deployment in resource-limited settings to address these barriers.

## 5 Conclusions

In this study, we developed SiameseNet, a dual-branch deep neural network incorporating Multi-Instance Learning and cross-attention mechanisms, to predict the histological grade of ICC using CT images. Our method demonstrated superior performance compared to traditional and transformer-based models, achieving an accuracy of 86.0% and AUC of 86.2% on the testing set. These results highlight the potential of our approach to mitigate the impact of tumor heterogeneity and improve diagnostic precision. This method could serve as a valuable tool in clinical practice, aiding timely and personalized treatment planning for ICC patients. Future work will focus on validating the model across multiple centers and enhancing its interpretability to facilitate clinical adoption.

## Data Availability

The data that support the findings of this study are available on request from the corresponding author.
